# Transcriptomic profiling and functional prediction reveal aberrant expression of circular RNAs during osteogenic differentiation in human umbilical cord mesenchymal stromal cells

**DOI:** 10.1038/s41598-021-98470-2

**Published:** 2021-10-06

**Authors:** Cheng Su, Xiao Zheng, Yanjin He, Li Long, Wenchuan Chen

**Affiliations:** State Key Laboratory of Oral Diseases, National Clinical Research Center for Oral Diseases, West China Hospital of Stomatology, Department of Oral Prosthodontics, Sichuan University, Sichuan, 610041 China

**Keywords:** Genome informatics, Differentiation, Stem-cell research

## Abstract

Circular RNAs (circRNAs) are crucial elements of non-coding RNA, that regulate various biological processes. To date, expression patterns and functional roles of circRNAs during osteogenic differentiation of human umbilical cord mesenchymal stromal cells (hUCMSCs) remain unknown. In this study, we analyzed RNA-sequence data to reveal expression profiles of circRNAs during osteogenesis of hUCMSCs, then elucidated the underlying mechanisms of action. We identified a total of 5457 circRNAs in hUCMSCs, of which 34 and 33 were upregulated and downregulated, respectively. We applied Gene Ontology and Kyoto Encyclopedia of Genes and Genomes analyses to determine functions and related pathways of differentially expressed circRNAs. Moreover, we applied bioinformatics tools to construct competing endogenous RNA networks, comprising 10 circRNAs, 46 micro RNAs and 413 mRNAs. Furthermore, we predicted protein-coding potential of the upregulated circRNAs then constructed a co-expression network comprising the top 5 upregulated circRNAs and 75 RNA-binding proteins. Next, we validated 6 differentially-expressed circRNAs and found that overexpressing circ‐CTTN could promote osteogenesis of hUCMSCs. Overall, our findings indicate that clusters of circRNAs are aberrantly expressed in hUCMSCs during osteogenic differentiation, hence lay a foundation for future research into promoting hUCMSCs osteogenic differentiation and bone regeneration.

## Introduction

Mesenchymal stromal cells (MSCs), which are characterized by multipotency characteristics, are found in many human tissues, such as bone marrow, skin, adipose and umbilical cord blood^1^. For years, bone marrow mesenchymal stromal cells (BMSCs) have been extensively studied for the reconstruction and regeneration of damaged tissues^2^. However, BMSCs’ application has been constrained by the invasive harvest procedure^3^. Thus, it is necessary to identify alternative sources of isolating MSCs. hUCMSCs, which can be easily collected from umbilical cord tissues of newborn infants, have been regarded as a favorable seed cell. In addition, these cells exhibit similar proliferation and osteogenic differentiation potential with those of BMSCs^4,5^. In fact, a recent study reported that scaffolds seeded with hUCMSCs cell aggregated successfully leading to formation of periodontal hard tissues^6^. Therefore, hUCMSCs have become an attractive option for use in bone tissue engineering. However, the precise molecular mechanism underlying osteogenic differentiation process by these cells is poorly understood.

CircRNAs, which have covalent and closed loop structures, are a novel type of non‐coding RNAs. This group of RNAs cannot be easily degraded by RNase R and possess higher stability than linear RNAs^7^. Accumulating evidences have indicated that circRNAs participate in multiple biological processes, including cell proliferation, apoptosis, cell differentiation, and tumorigenesis^8,9^. Furthermore, knocking down of circRNA CDR1as reportedly inhibited osteogenic differentiation of hUCMSCs^10^. However, the role of circRNAs regulatory network in hUCMSCs osteogenic differentiation process remains unknown, necessitating further explorations.

In the present study, we used high-throughput RNA sequencing technology to analyze expression profiles of circRNAs in hUCMSCs during osteogenesis. Results revealed various differentially expressed circRNAs, some of which were closely associated with hUCMSCs differentiation, and further showed potential functions after bioinformatics analysis. In addition, overexpression of circ-CTTN was found to promote osteogenic differentiation of hUCMSCs. Taken together, these findings indicate provide novel insights into understanding of osteogenic differentiation of mesenchymal stromal cells, and are expected to improve our knowledge of the circRNA modulation during osteogenic differentiation of hUCMSCs.

## Materials and methods

All materials and methods were performed in accordance with the relevant guidelines and regulations. Ethical approval was obtained from the ethics committee of Sichuan University. Informed consent from sample providers were signed.

### Cell cultures

hUCMSCs were supplied by the Sichuan Stem Cell Bank, Chengdu, China. The cells were cultured in 25 cm^2^ culture dishes with Dulbecco's minimum essential medium (DMEM; HyClone, USA), supplemented with 10% fetal bovine serum (FBS; GIBCO, USA) and 1% penicillin/streptomycin. The cultures were incubated at 37 °C in a humidified incubator maintained under 5% CO_2_. The medium was changed every other day, and the cells were passaged using 0.25% trypsin (HyClone, USA) after reaching 70–80% confluence. Cells at the 4th passage were used in the subsequent experiments.

### Osteogenic differentiation of hUCMSCs

hUCMSCs were seeded into six-well plates, at a density of 5 × 10^5^ per well. Upon attaining a 70–90% confluent, the cells were cultured in osteogenic induction medium [complete DMEM medium, dexamethasone (0.1 mM), ascorbic acid (50 ug/ml) and β-glycerolphosphate (10 mM) (all from Sigma-Aldrich)], while standard controls were cultured in complete medium.

### Alkaline phosphatase (ALP) assay and ALP staining

Alkaline phosphatase activity in the cells was assayed using the ALP assay kit (Beyotime, China), according to the manufacturer’s instructions. Briefly, cells were allowed to differentiate for 1 week, followed by addition of ALP reaction buffer containing p-nitrophenyl phosphate (pNNP), and ALP hydrolyzed colorless pNPP to yellow p-nitrophenol under optimal pH and temperature. Thereafter, ALP activity was determined by measuring absorbance of the reaction product. ALP activity was normalized to total cellular protein content, and assessed by BCA Protein Assay Kit (Beyotime, China) according to the manufacturer’s instructions. ALP staining was carried out using an ALP staining kit (Beyotime, China). Briefly, hUCMSCs were stained with NBT/BCIP solution at room temperature for half hour, washed with double-distilled water then observed under a microscope.

### RNA extraction and quantitative real-time polymerase chain reaction (qRT-PCR)

Total RNA was extracted from hUCMSCs, after 7 days of culture on osteogenic and non-osteogenic media, using TRIzol (Invitrogen, USA) according to the manufacturer’s instructions. RNA quantity and quality were assessed using a NanoDrop ND-1000 (NanoDrop, USA), and only RNA samples with absorbance 260/280 ratios between 1.8 and 2.1 used for further analysis. RNA integrity was checked by agarose formaldehyde gel electrophoresis.

Osteogenesis-related gene ALP, runt-related transcription factor 2 (RUNX2), and osteocalcin (OCN) were examined to verify the degree of osteogenic differentiation. Among the differentially expressed circRNAs, we randomly selected six circRNAs, namely circ_0004726, circ_0127664, circ_0003456, circ_0002607, circ_0000918 and circ_0003376, for qRT-PCR. The extracted total RNAs from both induced samples and control samples were reverse transcribed to complementary DNA (cDNA) using the RevertAid First Strand cDNA Synthesis Kit (Thermo Fisher, USA), then subjected to qRT–PCR in a LightCycler96 System (Roche) using Hieff TM qPCR SYBR® Green Master Mix (YEASEN, China). Amplification was performed under the following conditions: initial denaturation at 95 °C for 300 s, followed by 40 cycles of 95 °C for 10 s, annealing at 60 °C for 20 s, and extension at 72 °C for 20 s. Melting curve analysis was performed to verify non-specific amplification, and expression levels normalized to those of GAPDH used as an endogenous control.

### Transcriptome high-throughput sequencing

Total RNA was extracted from six samples using Trizol, then rRNAs removed using the Ribo-Zero® rRNA Removal Kit (Illumina, USA) according to the manufacturer’s instructions. RNA libraries were constructed using the TruSeq® RNA Sample Preparation Kit (Illumina, USA), then subjected to an Agilent 2100 Bioanalyzer system (Agilent Technologies, USA) for quality and quantity control. Quantified libraries were subjected to 150 bp paired-end sequencing on the HiSeq 4000 platfrm RapidRun (Illumina, USA) at a final concentration of 10 pM, and quality controlled by Q30. Low quality reads and adaptors were removed and trimmed, respectively, then the reads mapped onto hg19 using STAR genome (https://github.com/alexdobin/STAR). We employed the DCC software to identify circular RNAs, then annotated them using circbase database (http://www.circbase.org/). Moreover, we performed a hierarchical clustering analysis and generated volcano plots using TBtools^11^.

### GO enrichment and KEGG pathway analysis

To explore the main biological functions of the differentially expressed circRNAs, we performed functional enrichment analysis using gene ontology (GO), targeting molecular function, biological processes and cellular components. Meanwhile, we applied the KEGG pathway analysis to annotate and identify significantly upregulated pathways.

### Construction a circRNA-miRNA-mRNA network

Next, we used miRanda (http://www.microrna.org/microrna/home.do) and TargetScan (http://www.targetscan.org/vert72/) to predict the binding sites for human miRNAs within the top 10 upregulated circRNAs and the potential miRNA targets. Thereafter, we generated a competing endogenous RNA network using Cytoscape software.

### Prediction for protein-coding potential and interaction propensity of circRNAs

We adopted the catRAPID software (http://service.tartaglialab.com/page/catra pid_group) to predict the interaction propensity of top 5 circRNAs with RNA-binding proteins (RBPs). Thereafter, we predicted protein-coding potential of 34 significantly upregulated circRNAs using getorf (http://embossgui.sourceforge.net/demo/getorf.ht ml), then detected the potential open reading frames (ORFs) of query sequences using the same program according to the parameters set by the user.

### Vector construction and cell transfections

Sequence data revealed presence of 34 differentially upregulated circRNAs, among which upregulation of circRNAs, and circ-CTTN (hsa_circ_0003376) were validated by qRT-PCR in induced group. Therefore, we selected it for subsequent experiments, and synthesized a recombinant adenovirus vector encoding circRNA-CTTN by Hanbio Biotechnology (Shanghai, China). Prior to transfection, hUCMSCs were incubated in DMEM without FBS and antibiotics for 6 h, then infected with circ-CTTN-expressing adenovirus particles while control adenovirus at a multiplicity of infection of 100 for 6 h. The cells were maintained in the osteogenic induction medium for 7 days, after which RNA was extracted and subjected to qRT-PCR to confirm transfection success and examine levels of osteogenesis-related genes. ALP staining was also performed at day 7.

### Statistical analysis

All experiments were repeated at least three times, with all data presented as means ± standard deviations (SD) of the mean. Data Comparisons between and among groups were performed using a students’ *t*-test and analysis of variance (ANOVA) with Tukey–Kramer test, respectively. For all analyses, data followed by *P* < 0.05 were considered statistically significant.

## Results

### Osteogenic differentiation of hUCMSCs

**Figure 1:**
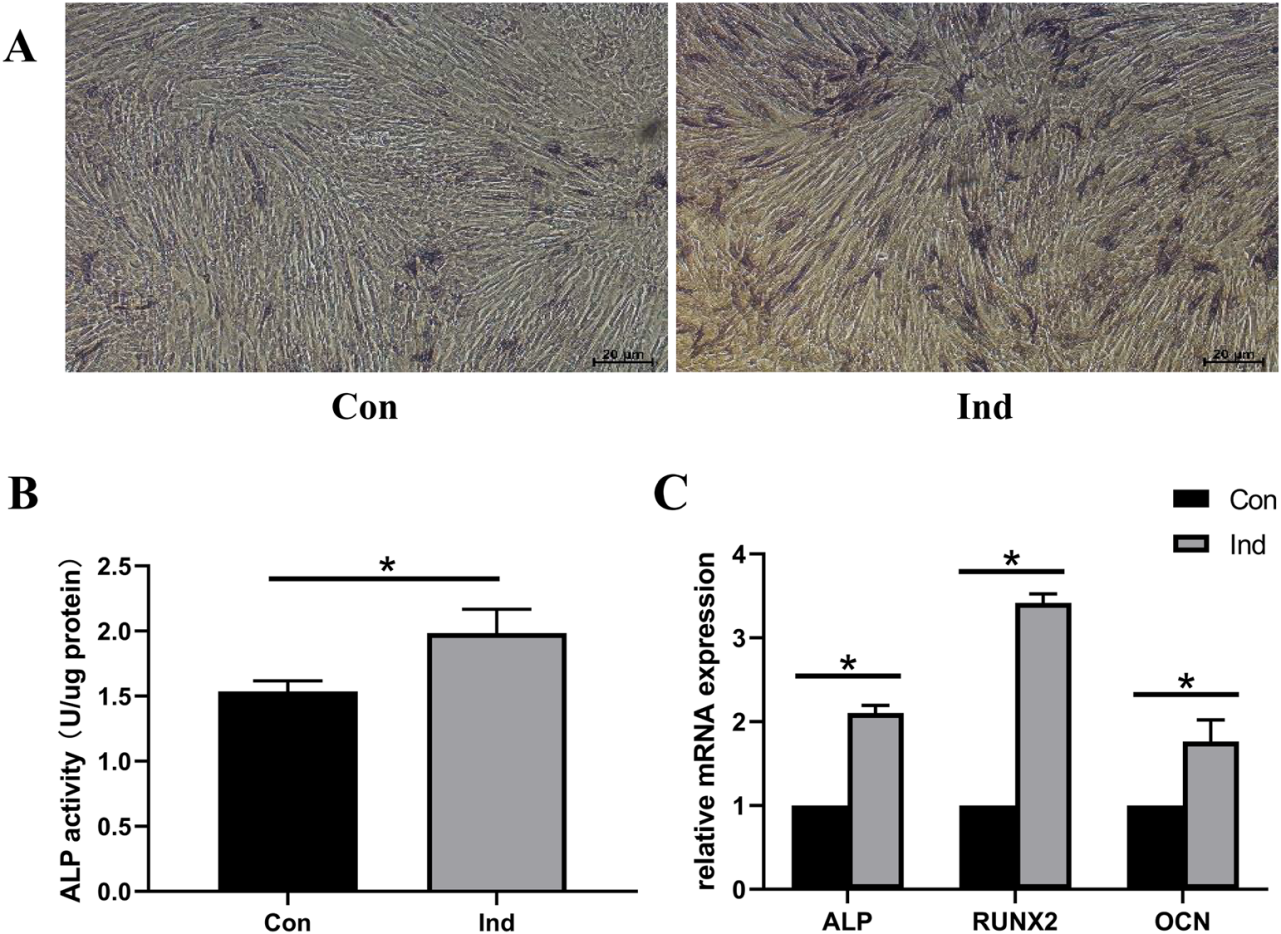
Profiles of osteogenic differentiation in hUCMSCs after osteogenic induction. (**A**) Images of ALP staining under microscopy. (**B**) ALP activity of the induced relative to control group. (**C**) expression profiles of ALP, RUNX2 and OCN in hUCMSCs after osteogenic induction for 7 days based on qRT-PCR. Ind: induced group; Con: control group.

hUCMSCs exhibited a significantly pronounced ALP staining, after osteogenic induction for 7 days (Fig. 1A), while ALP activity was significantly higher in the induced than the control group (Fig. 1B). In addition, osteogenesis-related genes, including ALP, RUNX2, OCN were significantly upregulated in induced group (Fig. 1C). These results represented osteogenic differentiation of hUCMSCs was successfully induced.

### Expression profiles of circRNAs in hUCMSCs and qRT‐PCR Validation

**Figure 2:**
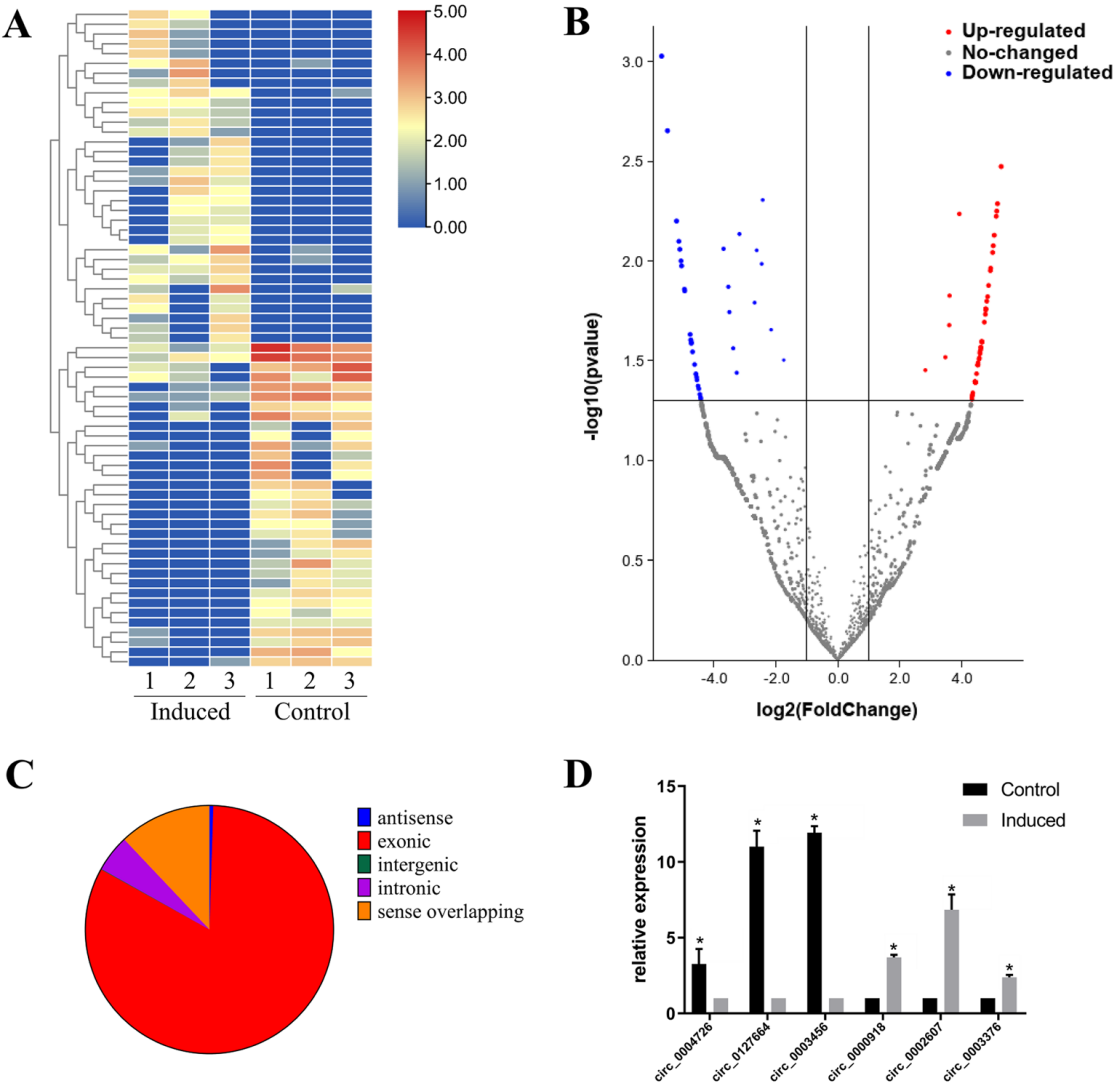
Differential expression of circRNAs in hUCMSCs. (**A**) A heatmap showing patterns of circRNAs expression between the induced and control groups. (**B**) A volcano plot showing profiles of circRNA expression based on fold changes and P-values. (**C**) Differentially expressed circRNAs classified using a pie chart. (**D**) Expression patterns of six selected circRNAs during osteogenic differentiation.

Transcriptomic analysis of hUCMSCs induced toward osteogenic differentiation revealed a total of 5,265 circRNAs in six samples. After filtering, excluding circRNAs which are only detected in one of six samples, 34 and 33 of them significantly upregulated and downregulated (fold change > 2.0; *P* < 0.05), respectively. Hierarchical clustering and a volcano plot showed that expression levels of circRNAs of the experimental group differed from those in the control group (Fig. 2A and B). The current research suggest that different circRNAs have different back splicing mechanisms and functions. Intronic and exon intron circRNAs can associate with U1 small nuclear ribonucleic proteins, then the complex affects RNA polymerase II activity to promote their parent gene expression in the nuclei^12^. In this experiment, we classified the circRNAs from hUCMSCs and found 4,347 exonic circRNAs, 253 intronic circRNAs, 4 intergenic circRNAs, 26 antisense circRNAs, and 635 sense overlapping circRNAs (Fig. 2C). Six of the significantly altered circRNAs were validated using qRT‐PCR. Particularly, circ_0002607, circ_0000918 and circ_0003376 were significantly upregulated in the experimental group, corroborating RNA-Seq results. Similarly, circ_0004726, circ_0127664, and circ_0003456 were significantly downregulated after qRT‐PCR (Fig. 2D).

### GO functional and KEGG pathway analyses

**Figure 3:**
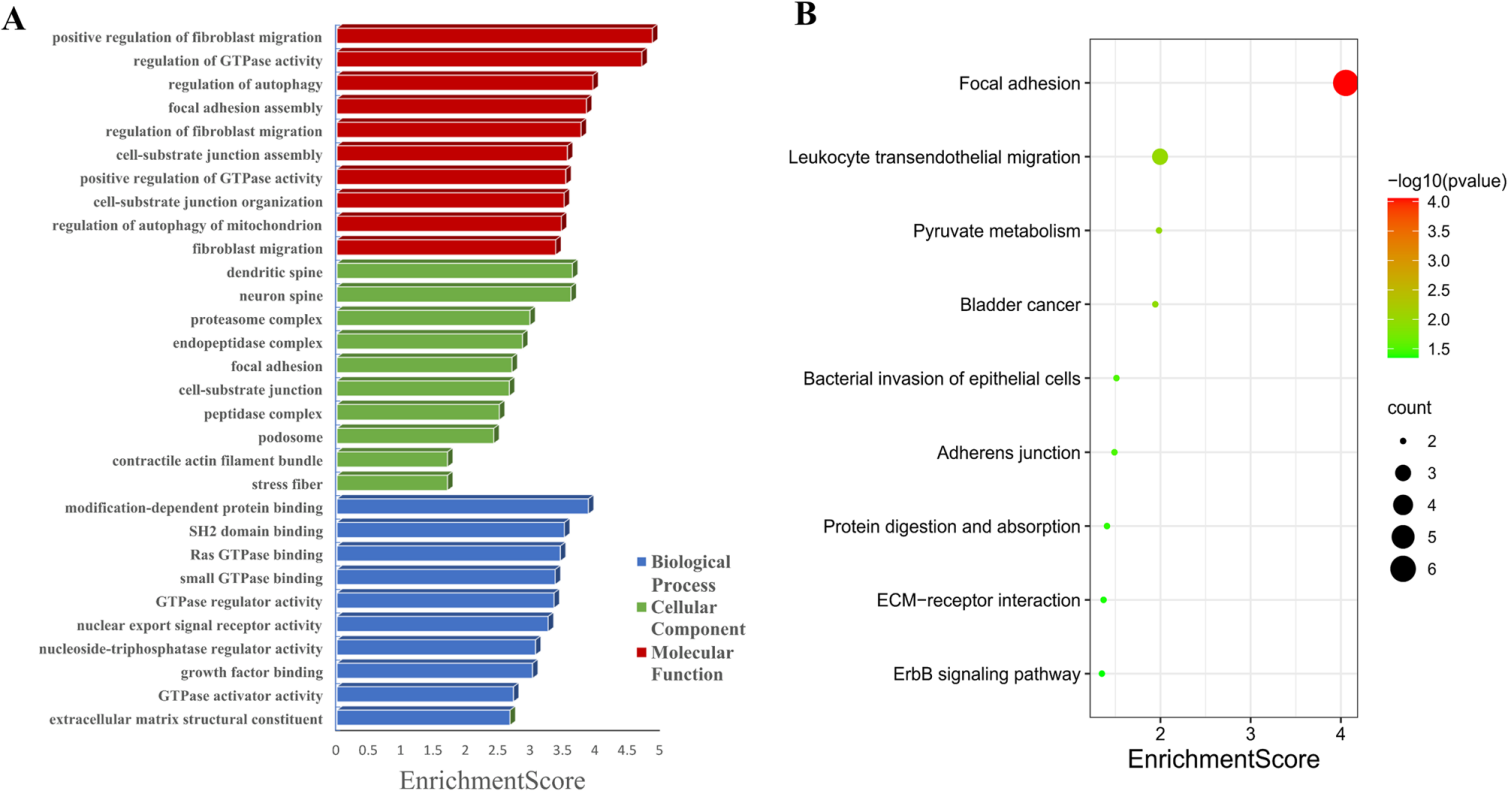
GO terms and KEGG pathways analysis of parental genes of circRNA in hUCMSCs. (**A**) GO term categories for differentially expressed circRNAs during osteogenic differentiation of hUCMSCs. The top 10 GO categories are shown. (**B**) KEGG pathway analysis of differentially expressed circular RNAs in hUCMSCs.

GO and KEGG pathway enrichment analyses were performed for parental genes of differentially expressed circRNAs. Results showed the different GO terms (Fig. 3A). With regards to biological processes, the most enriched GO terms were related to positive regulation of fibroblast migration, regulation of GTPase activity and regulation of autophagy. With regards to cellular components, these circRNAs were mainly involved in dendritic spine, while for molecular function, the most enriched GO term was modification-dependent protein binding. KEGG pathway analysis revealed significant enrichment in 9 pathways. Specifically, the most enriched pathways were focal adhesion, leukocyte transendothelial migration and pyruvate metabolism (Fig. 3B).

### Analysis of circRNA-miRNA-mRNA network

**Figure 4:**
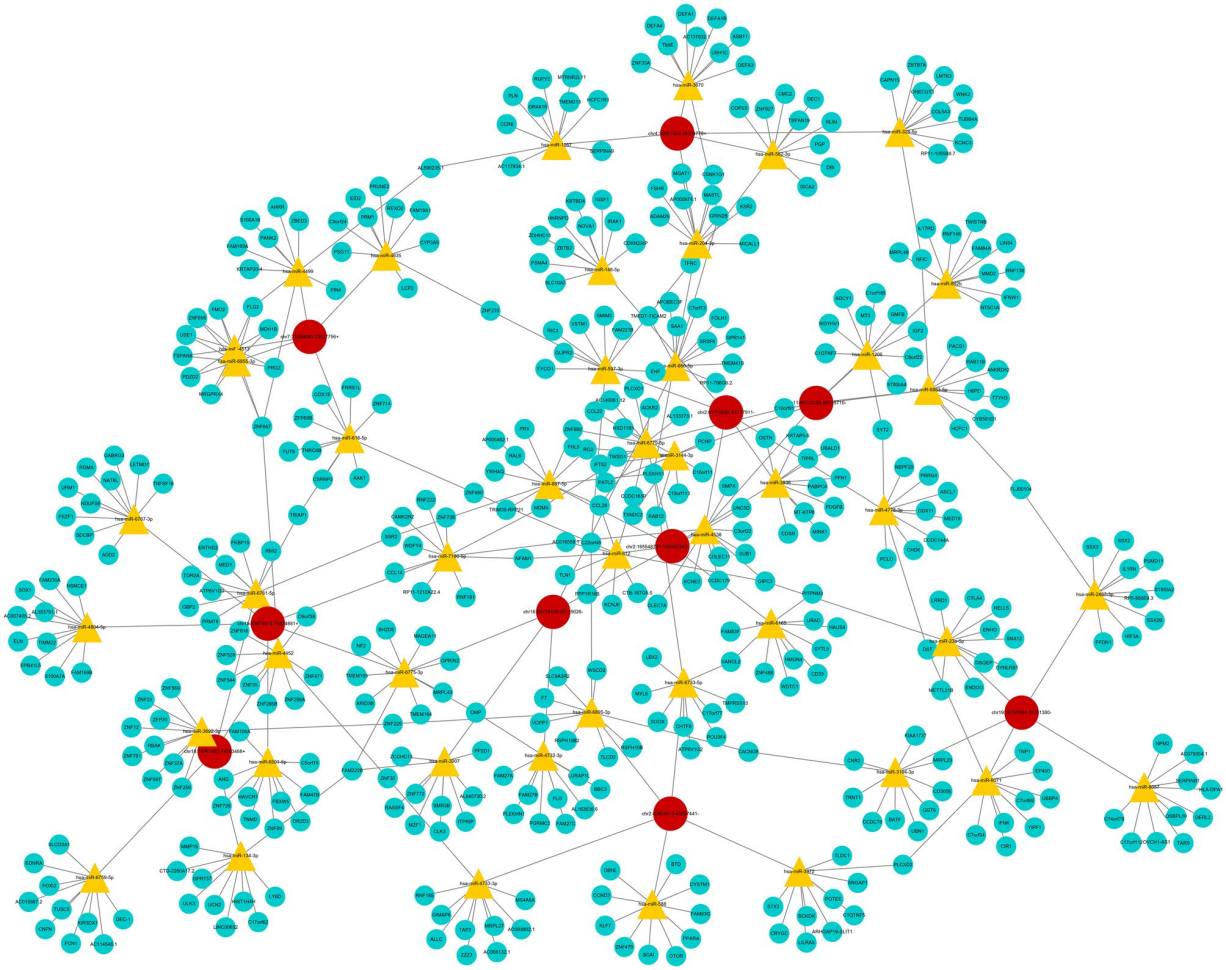
A CircRNA-miRNA-mRNA network map of differentially expressed circular RNAs based on bioinformatic prediction. Red circular nodes represent circRNAs, yellow triangle nodes denote miRNAs while blue circular nodes represent mRNAs.

To further investigate the potential functions of circRNAs, we constructed a circRNA‐miRNA‐mRNA network using our RNA‐seq data. In the illustration, each circRNA connected with five high‐binding potential miRNAs whereas each miRNA connected with ten high‐binding potential mRNAs (Fig. 4). Moreover, the network revealed an interaction between 10, 46, and 413 kinds of circRNAs, miRNAs, and mRNAs, respectively. For example, miR-4538 interacted with 3 circRNAs and 10 mRNAs, to participate in a variety of metabolic pathways during hUCMSCs osteogenic differentiation. Overall, these results indicated that the 10 circRNAs might be closely associated with gene regulation during osteogenic differentiation of hUCMSCs.

### Computational prediction of circRNA protein-coding and protein interaction

**Figure 5:**
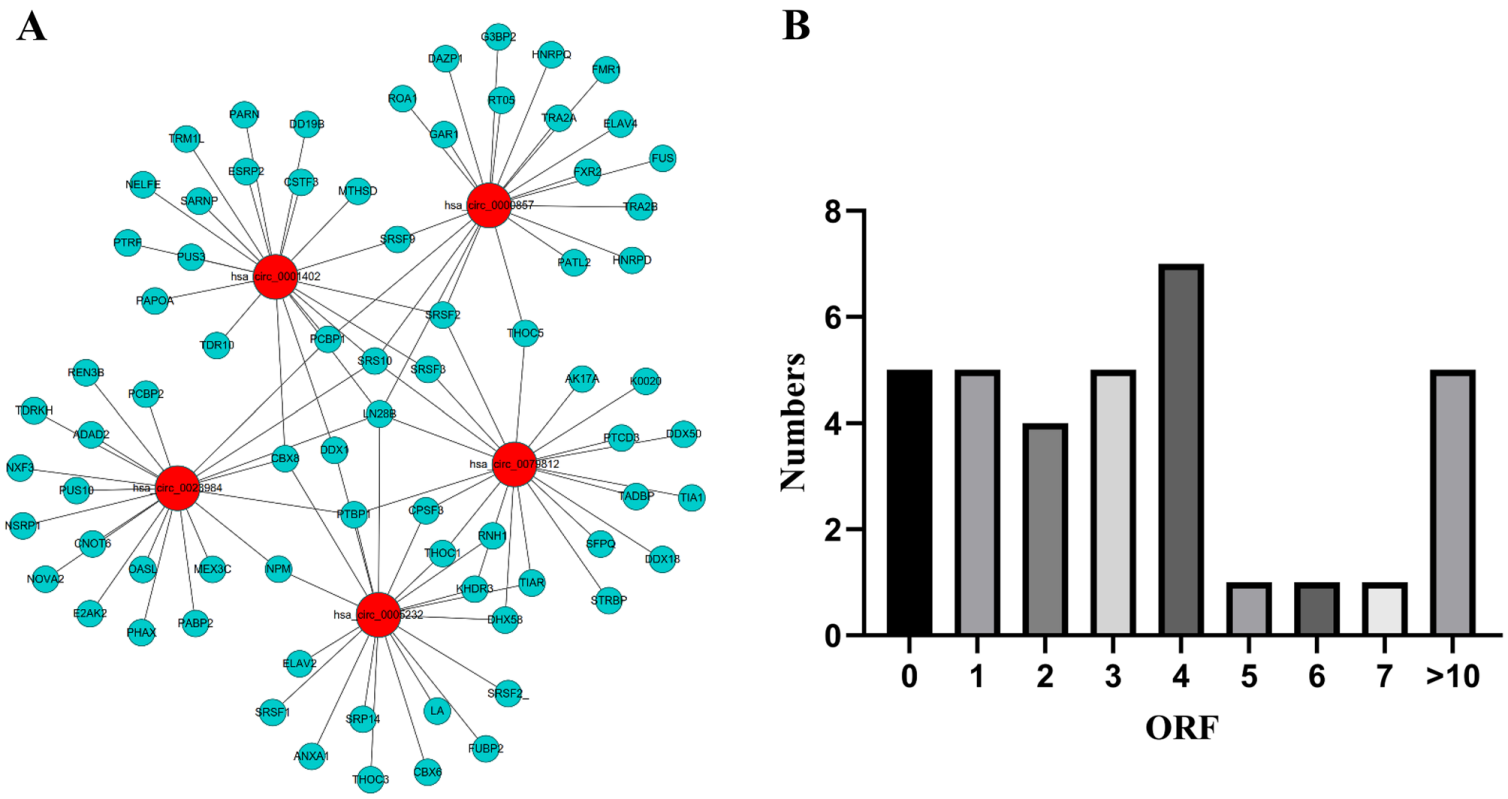
A co-expression network indicating predicted RBPs and protein-coding potential of upregulated circRNAs. (**A**) CircRNA-protein co-expression network based on predicted interactions. Red and blue circular nodes represent circRNAs and RBPs, respectively. (**B**) Predicted ORFs in circRNAs.

To identify important RNA-binding proteins interacting with circRNAs, we correlated profiles of circRNA expression with RBPs using the catRAPID database. Results indicated that for each of top 5 upregulated circRNAs analyzed, there was a broad range of interactions between circRNAs and a large number of RBPs. In total, we identified 100 pairs of significant co-expression relationships between 5 differential circRNAs and RNA-binging proteins. Then, we constructed a circRNA-protein co-expression network based on the predicted interactions (Fig. 5A).

Next, we evaluated the protein-coding potential of the circRNA, then predicted open reading frames in the differentially upregulated circRNAs using Getorf. Any frame with a length over 30 bases was considered an ORF. Based on this, we calculated the length and number of ORFs in 34 circRNAs which are significantly upregulated. As shown in Fig. 5B, we found 5 circRNAs with no ORF, 5 had 1, 4 had 2, 5 had 3, 7 had 4, and 8 had 5 or more ORFs.

### Overexpression of circ-CTTN promotes osteogenic differentiation of hUCMSCs

**Figure 6:**
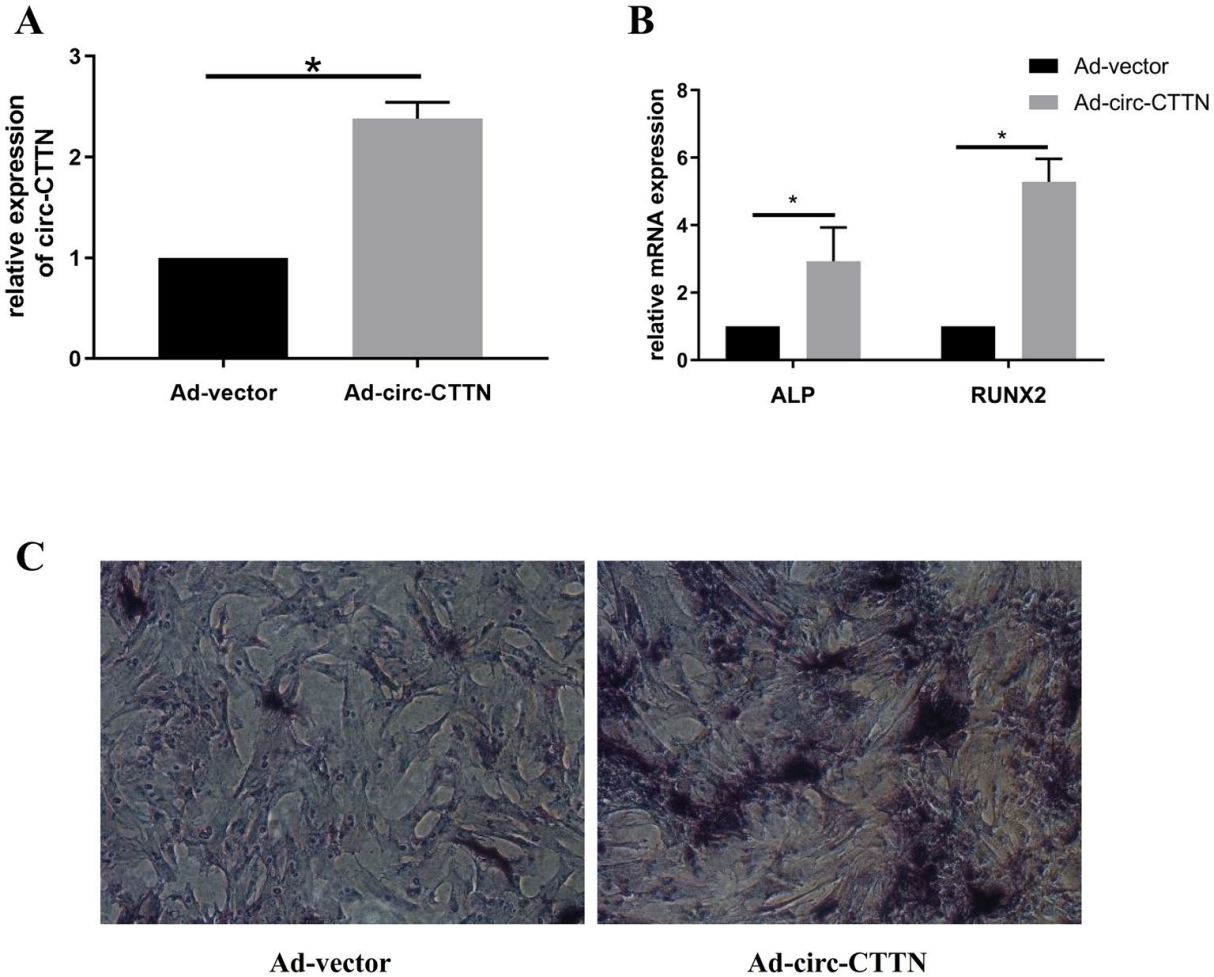
Profiles of Circ-CTTN in promoting osteogenic differentiation of hUCMSCs. (**A**) qRT-PCR confirm successful overexpression of circ-CTTN in hUCMSCs. (**B**) Upregulation of ALP and RUNX2 mRNAs in hUCMSCs after overexpression of circ-CTTN. (**C**) Images of ALP staining under microscopy.

To ascertain circRNAs’ regulatory role, we overexpressed circ-CTTN in hUCMSCs cells via transfection and found a significant increase of circ-CTTN in the overexpression group (Fig. 6A). Results of qRT-PCR revealed significant upregulation of ALP and RUNX2 mRNAs in the circ-CTTN overexpression group (Fig. 6B), while ALP staining indicated increased differentiation in the circ-CTTN overexpression group after 7 days of induction (Fig. 6C). These results affirmed that circ-CTTN promotes osteogenesis of hUCMSCs.

## Discussions

HUCMSCs are expected to become important seed cells for bone tissue engineering, since they can be easily obtained and are their application is not limited by ethical concerns. However, the exact mechanism underlying osteogenic differentiation of the hUCMSCs remain unclear. Results from recent studies have uncovered the functions of circRNAs, while circRNA expression profiles have been characterized across multiple tissues or cells. For example, RNA sequencing revealed a total of 1,456 circRNAs in periodontal ligament stem cells, while the osteogenic differentiation was found to be regulated by circRNA BANP as a molecular sponge for miRNAs^13^. Similarly, Li et al.^14^ obtained 333 and 317 upregulated circRNAs and downregulated circRNAs, respectively, during osteogenic differentiation of stem cells from apical papilla.

In the present study, we used high-throughput RNA sequencing and bioinformatics to assess link between circular RNAs and osteogenic differentiation of hUCMSCs, and obtained a total of 5,265 circRNAs of which 3,941 were annotated in circBase. Among them, 34 and 33 circRNAs were upregulated and downregulated, respectively. Consequently, we randomly selected 6 circRNAs from RNASeq and validated their expression via qRT-PCR.

Previous studies have demonstrated that circRNAs have a variety of functions, such as miRNA sponge activity, binding with RBPs and influencing the expression of parental genes^15^. Recently, results from functional studies showed that some circRNAs harbor miRNAs binding sites and block the functional roles of miRNAs. For instance, the circRNA CDR1as, which is highly expressed and conserved in cells, contains more than 70 miR-7-binding sites hence it can act as a sponge for miR-7 and strongly inhibit miR-7 activity, thereby modulating expression of miR-7 target gene expression^16^. In the present study, we predicted the interaction between circRNA-miRNA networks and found that osteogenesis-related miRNAs, such as miR-612, miR-146a and miR-204 were involved in the network. Previous evidences have shown that miR-612 was downregulated during osteogenic differentiation in human adipose‐derived stem cells^17^, whereas miR‐204 can inhibit osteogenesis of BMSCs by acting as a negative regulator of Runx2^18^. Our constructed mRNA‐miRNAs co-expression networks revealed some osteogenic-related genes, such as NFIC and OCN, have been identified^19,20^. These findings indicate that the sponge function of circRNAs serve an important role in the osteogenic differentiation of hUCMSCs.

Next, we predicted the RBP binding sites of differentially expressed circRNAs. Particularly, RBPs play crucial roles in various cellular processes, such as regulating RNA splicing, polyadenylation, stability, localization, translation, and degradation^21^. Our circRNAs-RBPs co-expression network revealed that protein LIN28B could interact with all five circRNAs. A previous study demonstrated that Lin28B is a highly conserved RBP and numerous which has further been found to contribute to maintenance of the stemness phenotype in stem cells and cancerous cells^22^. Notably, that LIN28B was found to inhibit biogenesis of miRNA let-7, which in fact proved to enhance osteogenesis of human mesenchymal stromal cells^23,24^. Moreover, our results showed that SRSF2, PCBP1, PTBP1 and CBX8 were the key RBPs with three interactions with circRNAs. Furthermore, the protein-circRNA interaction network indicated the linkage between circRNAs and protein in osteoblast differentiation.

Accumulating evidences have shown that circRNAs are not just accidental by-products of mis-splicing, and some of them exhibit translation activity. In fact, previous reports have revealed existence of a 753 nt ORF in circZNF609, which can be translated into a polypeptide^25^. Noteworthy, results from a previous research showed that a single N6-methyladenosine circRNA site was sufficient to promote cap-independent translation of circRNAs by recruiting initiation factor eIF4G2^26^. Apart from presence of ORFs, translation of circRNAs relies on regulatory elements, such as internal ribosome entry site, m6A-modified conserved sites and rolling cycle amplification^26,27^. In the present study, we detected 29 circRNAs that had ORFs, which might be potential candidates for encoding proteins. However, further studies are needed to verify the reliability of these sites and establish whether the circRNA sequence can be translated into proteins.

Results from qRT-PCR confirmed a significant increase in circ-CTTN in induced group. Therefore, we further investigated whether circ-CTTN impacts osteogenic differentiation and found that overexpressing this factor significantly promoted osteogenic differentiation of hUCMSCs. Circ-CTTN is derived from the CTTN (cortactin) gene exon 1–3, with a spliced mature sequence length of 258 bp. Previous studies have shown that cortactin can be considered a potential prognostic marker in cancer^28,29^, and also plays an essential role in osteoclast actin assembly as well as bone resorption^30^. Although preliminary results indicated that circ-CTTN plays an important regulatory role in hUCMSCs, the specific mechanisms underlying its action remain unknown necessitating further explorations.

Although findings of this study may have potential implications in guiding bone tissue engineering, several limitations should be noted. Firstly, the circRNAs were identified at the early, but not late phase of osteoblast differentiation. Secondly, we used a small sample size, hence future studies should consider larger samples to improve the accuracy. Thirdly, our analysis of circRNA protein-coding and protein interaction abilities were performed via a single prediction approach, although multiple approaches may provide more accurate predictions.

## Conclusion

Despite the aforementioned limitations, our findings clarify the underlying mechanism of osteogenic differentiation in hUCMSCs. Specifically, analysis of high-throughput RNA sequencing revealed aberrantly expressed circRNAs and their genome distribution. Further analysis revealed 34 and 33 upregulated and downregulated circRNAs, respectively in the hUCMSCs during osteogenesis. Furthermore, a constructed circRNA-miRNA-mRNA regulatory network as well as results from bioinformatics analysis predicted protein-coding potential and RBPs binding activity of circRNAs. Taken together, these findings provide new insights into the role of circ-CTTN in osteogenic differentiation of hUCMSCs, while profiles of circRNA expression enhance our understanding into the regulation of osteogenic differentiation.

## Supplementary Information

Supplementary Information 1.

Supplementary Information 2.
