# Supplementary material for: Transcriptomic profiling and functional prediction reveal aberrant expression of circular RNAs during osteogenic differentiation in human umbilical cord mesenchymal stromal cells
Source: Sci Rep. 2021 Oct 6;11:19881. doi: 10.1038/s41598-021-98470-2 (PMC8494929; doi:10.1038/s41598-021-98470-2)
Supplement: Supplementary file 1 — Supplementary Information 1. [file 41598_2021_98470_MOESM1_ESM.docx]

Table. The detailed characteristics of qRT-PCR for mRNAs and circRNAs

| Target name | Primer sequence |
| --- | --- |
| ALP | F :5' AGCACTCCCACTTCATCTGGAA 3'  R :5' GAGACCCAATAGGTAGTCCACATTG 3' |
| RUNX2 | F :5' TCCACACCATTAGGGACCATC 3'  R :5' TGCTAATGCTTCGTGTTTCCA 3' |
| OCN | F :5' CCACTGCTTCTCCATCAAGGGTC 3'  R :5' GGTCAGCCAACTCGTCACAGTC 3' |
| hsa_circ_0002607 | F :5' CGGCTCTGAGAAGGATGA 3'  R :5' GCTTGGAGGTACTGGACT 3' |
| hsa_circ_0003376 | F :5' GAATGATGTGAGTGAGAAGG 3'  R :5' AACACAGTAATCCAATGAGG 3' |
| hsa_circ_0000918 | F :5' TTCTGGAGCACGATCTCA 3'  R :5' AAGCAGTTCCTTGGCATAG 3' |
| hsa_circ_0004726 | F :5' GTCCTCCAGACCATCTATAAG 3'  R :5' CTCTGACCACCTCTGTACT 3' |
| hsa_circ_0127664 | F :5' TTCACTGATGCCTCCTCTA 3'  R :5' AGTCATCTGCTCCATTGTC 3' |
| hsa_circ_0003456 | F :5' CTACATCGCCAGTCTGAAG 3'  R :5' CTCCACACCAAGAACAACA 3' |
| GAPDH | F :5' GGCCTCCAAGGAGTAAGACC 3'  R :5' AGGGGAGATTCAGTGTGGTG 3' |
